# AGE-SPECIFIC POPULATION ATTRIBUTABLE FRACTIONS FOR THE IMPACT OF HYPERTENSION ON INCIDENT DEMENTIA

**DOI:** 10.1093/geroni/igad104.1293

**Published:** 2023-12-21

**Authors:** Jason Smith, A Richey Sharrett, James Pike, Rebecca Gottesman, Pamela Lutsey, B Gwen Windham, Josef Coresh, Jennifer Deal

**Affiliations:** Johns Hopkins Bloomberg School of Public Health, Baltimore, Maryland, United States; Johns Hopkins Bloomberg School of Public Health, Baltimore, Maryland, United States; Johns Hopkins University, Baltimore, Maryland, United States; NINDS/ NIH, Bethesda, Maryland, United States; University of Minnesota, Minneapolis, Minnesota, United States; UMMC-The MIND Center, Jackson, Mississippi, United States; Johns Hopkins University, Baltimore, Maryland, United States; Johns Hopkins University, Baltimore, Maryland, United States

## Abstract

The population attributable fraction (PAF) of dementia in United States attributable to hypertension is estimated at 7-9%. However, prior studies of the PAF might underestimate hypertension’s impact on dementia risk, given specific design limitations. Additionally, prior work neither accounted for changing prevalence/effects of hypertension with age nor considered early vs. later occurring dementia. Here, we quantified PAFs of dementia by age 80 from hypertension measured at midlife and late-life using individual-level data from the Atherosclerosis Risk in Communities Study. Incident dementia was determined by adjudicated review, telephone interviews, informant interviews, hospitalization records, and death certificates. We used measures of systolic and diastolic blood pressure (SBP, DBP) to define BP categories: normal BP (SBP < 120 and DBP < 80), elevated BP (SBP 120-129 and DBP < 80), stage 1 hypertension (SBP 130-139 or DBP 80-89), and stage 2 hypertension (SBP ≥140 or DBP ≥90); we also defined an overall non-normal BP value (SBP ≥120 or DBP ≥80). We generated hazard ratios (HRs) of 32-year incident dementia from Cox regression models that adjusted for demographic and clinical characteristics, and stratified by age at BP ascertainment (45-54, 55-64, 65-74). We then used the HRs to quantify PAFs of dementia from hypertension. We estimated that 15-20% of dementia cases by age 80 were attributable to non-normal BP. The strongest PAFs (12-21%) were from stage 2 hypertension. Current estimates of the dementia risk attributable to hypertension may be low. Targeting hypertension from midlife through early late-life could sizably reduce dementia risk.

